# A Clinical Profile of Pediatric COVID-19 Testing in the Emergency Department, Dubai, United Arab Emirates

**DOI:** 10.1155/2022/5092259

**Published:** 2022-08-12

**Authors:** Fatima Farid Mir, Maysa Saleh

**Affiliations:** Pediatric Department, Latifa Women and Children Hospital, Dubai, UAE

## Abstract

**Background:**

The COVID-19 pandemic marked a health and economic crisis of massive proportions. In its early months, literature was centered on adult medical and critical care. As time progressed, international reports of COVID-19 infection in children steadily grew; however, data on disease features in the United Arab Emirates' pediatric population was noticeably lagging.

**Method:**

The presented research was conducted at Latifa Women and Children Hospital Emergency Department to ascertain an association between a child's presenting features and basic investigations to a subsequent positive COVID-19 test result. Data was collected via electronic medical records and statistical analysis performed with SPSS version 22.0.

**Results:**

A total of four hundred and five (405) patients were analyzed, with 32 (8%) being COVID-19 positive on initial testing in emergency department. There is a statistically significant correlation (*p* < 0.05) between testing positive for COVID-19 infection and history of exposure to COVID-19-positive individuals; the presence of runny nose, cough, poor feeding, and abdominal pain with reassuring physical examination findings; and predominantly normal reports of basic blood investigations and chest X-ray images.

**Conclusion:**

This research demonstrates that a minority of children tested for COVID-19 in the initial wave of the pandemic tested positive. A significant proportion of COVID-19-positive pediatric patients exhibit history of exposure to COVID-19-positive individuals; the presence of runny nose, cough, poor feeding, and abdominal pain; normal physical examination; normal basic blood investigations and chest X-ray findings.

## 1. Introduction

December 2019 marked the start of a global medical emergency, one which proved itself unique as no assured foresight could be made about the nature of COVID-19 infection in humans. The adult and elderly age groups were the hardest hit initially, and children seemed to be a more ambiguous group of the population.

Studies were swiftly issuing worldwide to navigate adult patient care; however, data on children lagged significantly and at most was in the form of case reports. A vague clinical spectrum of pediatric infections presented itself, and so the indications to test a child for COVID-19 infection were obscure.

SARS-CoV-2 infection was first brought to attention in December 2019 after clusters of pneumonia cases erupted in the Chinese city of Wuhan [1, 2]. Disease spread occurred at an alarming speed, reaching the scale of a global pandemic within a matter of weeks. As of 6th September 2020, there is an estimate of 26,763,217 infected cases, with 876,616 confirmed deaths and 216 countries affected by COVID-19 disease worldwide [1]. In United Arab Emirates, as of 19th September 2020, a total of 84,242 cases and 404 deaths have been recorded [3]. The number of cases has only increased exponentially since then, and patterns of infection evolved unpredictably with newer virus variants.

SARS-CoV-2 virus is believed to have originated from an animal source, with subsequent disease transmission primarily via airborne particles and contact with contaminated surfaces [2]. The rapidity of global infection spread has been confounding; however, much may be invariably attributed to the expansive reach of modern travel across cities and nations.

Clinical characteristics of COVID-19 are diverse and involve multiple organ systems at varying degrees [4]. Disease features in both neonates and children are under intensive investigation to delineate clinical course of SARS-COV-2 infection [5–7].

Childhood infection with SARS-CoV-2 may range from an asymptomatic individual to upper respiratory tract infection, skin rashes, pneumonia, gastrointestinal upset, acute respiratory distress syndrome, nervous system involvement, rhabdomyolysis, and the newly recognized Kawasaki disease like presentation of Multisystem Inflammatory Syndrome in Children [8–13].

A general pattern has been reported from multiple countries, revealing the fact that children have a much milder disease severity than adults, and scant reports are available on pediatric mortality secondary to COVID-19 [14]. Several hypotheses have been suggested to explain disease mildness in children, such as trained immunity secondary to immunization, presence of common viruses in their respiratory tracts that compete with SARS-CoV-2 for surface area, and the differences in expression of angiotensin-converting enzyme receptor 2 which is necessary for virus binding [15, 16].

The value of screening children from COVID-19 is being increasingly recognized, as asymptomatic or mild presentations may lead to neglected isolation precautions and subsequent dissemination of infection. Studies have also demonstrated prolonged fecal shedding of SARS-CoV-2 virus in children who have apparently recovered from COVID-19, raising the possibility of children acting as major sources of disease spread among communities [17]. Moreover, there are reports of children being coinfected with SARS-CoV-2 along with other viruses, which may have caused the presence of COVID-19 to be overlooked and subsequent isolation precautions neglected [8].

The presented research is aimed at shedding light on the clinical features of children tested for COVID-19 infection and their subsequent test outcome, with an underlying target of guiding testing in children. If a significant relation can be made between a child's complaint and eventual COVID-19 result, pediatricians can be alerted to these trends and be better able to judge which child needs a COVID-19 test. Judicious testing enables superior hospital resource utilization as unnecessary testing would be reduced, and key disease features can be sought out to avoid missing the opportunity to diagnose a child who is COVID-19 positive. Moreover, international data on pediatric disease features may not be a true reflection of the patterns displayed by children in the United Arab Emirates; hence, the importance of a local study cannot be overstated.

## 2. Materials and Methods

### 2.1. Study Design and Setting

The study was conducted in the setting of Latifa Hospital Pediatrics Emergency Department in Dubai, UAE, as this hospital was one of the largest pediatrics tertiary care facilities of Dubai Health Authority. This study is designed as a noninterventional, retrospective cross-sectional study.

### 2.2. Study Rationale

The underlying rationale of this research is to establish a comprehensive picture of the local pediatric population in the context of testing for COVID-19. This will help pediatricians navigate the testing process in children and hold a certain amount of confidence when judging which child is at a higher likelihood of eventually testing positive for COVID-19 infection. The research is justified in the light of ascertaining a relationship between the variables studied and a subsequent COVID-19 result.

### 2.3. Study Hypothesis

There is a statistically significant association between certain features of presenting history, physical examination, initial laboratory investigations, and chest X-ray findings to the COVID-19 test outcome.

### 2.4. Study Population

The target population is all pediatric patients who attended Latifa Hospital Emergency Department and underwent testing for COVID-19 infection via RT-PCR nasopharyngeal swab from January 1st to August 31st, 2020. The inclusion and exclusion criteria are elaborated as below:

The following are the inclusion criteria:

- All children from birth (zero years) to thirteen years old
- Both males and females included
- Symptomatic children of any severity who underwent testing for COVID-19
- Children who were diagnosed with surgical conditions however held a mixed etiology (may have infectious origin): acute appendicitis and skin abscesses
- Asymptomatic children who were brought for testing after close contact with a known COVID-19-positive individual or recent return from high-risk country (as determined by the World Health Organization guidance at the time of testing)
- Testing method via reverse transcriptase polymerase chain reaction (RT-PCR) nasopharyngeal swab or secretion aspirate for SARS-CoV-2 virus

The following are the exclusion criteria:

- Children beyond the age of thirteen years were not included in the study as this age group was not considered to be within the pediatric population in the hospital of study
- Children presented with exclusively surgical conditions (i.e., near absent likelihood of holding an infectious etiology): strangulated/incarcerated inguinal hernias; ovarian/testicular torsions; and intestinal malrotation/obstruction due to underling gut anomalies (hypertrophic pyloric stenosis, Hirschsprung disease, volvulus, intestinal atresia, etc.)
- Children attended with traumatic or environmental injuries: thermal/chemical burns, drowning/near-drowning, nonaccidental child abuse, bone fractures, skin lacerations, and animal/human bites
- Patients were electively screened for COVID-19 infection in the emergency department prior to elective preplanned admission for a nonemergency procedure: elective surgery, enzyme replacement therapy, and endoscopy/colonoscopy
- Second and subsequent swab results of children were tested multiple times for COVID-19 infection after hospital admission. Only the initial COVID-19 swab result from emergency department visit was included in the study, and the remainder of the tests once the child was admitted to the hospital were disregarded and excluded from the study
- Children who brought reports of COVID-19 result from an external non-Dubai Health Authority facility were not included as the reliability of the test results was doubtful and in repeated for confirmation before management decisions made in real time
- Patients presenting beyond the specified age, study time frame, and location were excluded as well

### 2.5. Sample Size and Sampling Procedure

After obtaining ethical approval, patient data was obtained from the hospital's electronic medical record system (“Salama” system) with absolute confidentiality and sensitivity to patient rights. A total of six hundred and twenty-nine (629) patients' records were obtained. Two hundred and twenty-three (223) patients were excluded based upon the exclusion criteria as aforementioned. All the remaining four hundred and five (405) patients successfully met the study's inclusion criteria and hence were fully retained for further data analysis and not randomly sampled out. The principal investigator handled patient data.

### 2.6. Data Collection Plan

Data was obtained from the hospital electronic medical records system management team (“Salama” team), who provided a list of patients who attended Latifa Hospital Emergency Department from January 1st to August 31st, 2020, and had the relevant International Classification of Disease (ICD) coding documented in their medical file (ICD-9: V73.99, V73.89 (“screening for viral diseases”) and ICD-10: Z11.59 (“encounter for screening for other viral disease”)). The “Salama” team had extracted these patients from their data base and provided the list in an excel sheet. The total number of patients obtained was six hundred and twenty-nine (629) as aforementioned and were narrowed down to four hundred and five (405) for statistical analysis and interpretation.

Variables studied in this research are as follows:

(i) Demographic data: patient age in years, gender, and nationality
(ii) Presence of a known COVID-19 exposure
(iii) Presence of a preexisting medical/surgical comorbidity
  - Presenting symptoms (runny nose, cough, poor feeding, difficulty breathing/apnea, vomiting/diarrhea, abdominal pain, head/throat/body pain, and skin rash)
  - Physical examination findings (congested ear, nose, throat (ENT); dehydration; lymphadenopathy; chest crackles/wheezes on auscultation; abdominal tenderness; skin rash; and joint or local tissue swelling)
  - Basic blood investigation results (white cell count (WBC); hemoglobin (Hb); absolute neutrophil count (ANC); absolute lymphocyte count (ALC); platelet count; c-reactive protein (CRP); procalcitonin; presence of hyponatremia and low serum bicarbonate)
  - Chest X-ray features (normal; nonspecific infiltrates; lobar consolidation; not performed); and finally, COVID-19 RT-PCR swab result (negative or positive)

### 2.7. Statistical Analysis

Statistical analysis was carried out using Excel software program and Statistical Package for the Social Sciences (SPSS) version 24.0. The following statistical analyses were carried out:

- Frequency tables, graphs, and descriptive statistics were performed
- Normal distribution of the continuous variables was assessed using the Kolmogorov-Smirnov test. Median and interquartile range (IQR) was used for the data that do not follow the normal distribution
- Categorical variables were presented as numbers and percent and were compared using the chi-square test. Fisher's exact test and Monte-Carlo exact test were used in case of small frequency
- Univariate logistic regression was used to investigate factors associated with positive COVID-19 test. The dependent variable is COVID-19 test (0 for negative, 1 for positive), and independent variables are demographic characteristics, morbidity, and history of contact, presenting symptoms, physical examination findings, chest X-ray results, and laboratory parameters. The results were presented by odds ratio (OR) and 95% confidence interval [18, 19]
- Stepwise multiple logistic regression was carried out to delineate the predictors of positive COVID-19 test, where the significant factors from the univariate logistic analysis were entered the model

The *p* values of <0.05 was the cut off level of significance.

## 3. Results

The presented study was conducted on a total of four hundred and five (405) patients who attended Latifa Hospital Emergency Department from January 1st to August 31st, 2020.

### 3.1. Patient Characteristics

Among the overall studied patient population: gender wise, females were of higher percentage than males (54.3% versus 45.7%). Non-UAE locals were more than UAE locals (64.0% versus 36.0%). The predominant age group was from 1 to <5 years (31.9%). Underlying comorbidities (such as prematurity, leukemia, genetic disorders, asthma, epilepsy, cerebral palsy, and congenital heart disease) were absent in 54.3%, and 19.5% have a known previous exposure to COVID-19 infection. Of the 405 patients studied, 32 (8%) tested positive and 373 (92%) tested negative for COVID-19 infection. Patient characteristics were compared according to COVID-19 swab test results with a highly statistically significant difference demonstrated between known exposure to COVID-19 infection and positive swab result (*p* value of 0.000).

### 3.2. Presenting Symptoms

The commonest presenting symptoms included fever (41.7%), vomiting/diarrhea (31.9%), and cough (21.5%). Each individual presenting symptom was subsequently studied according to COVID-19 swab test results. Patients with positive COVID-19 test results presented with the symptoms of fever (59.4%), runny nose (34.4%), cough (37.5%), poor feeding (3.1%), and abdominal pain (3.1%) as compared to those with a negative result (40.2%,10.2%, 20.1%, 16.9% and 16.9%, respectively), and the differences were statistically significant

### 3.3. Physical Examination Findings

A diverse range of physical examination features was observed, the commonest being congested ear/nose/throat (15.3%), dehydration (13.1%), and tenderness on palpation of the abdomen (11.4%). Each individual physical examination finding was subsequently studied in relation to COVID-19 swab outcomes; there is no statistically significant difference in all the examination findings between patients with negative or positive COVID-19 test

### 3.4. Laboratory Findings

**Figure 1:**
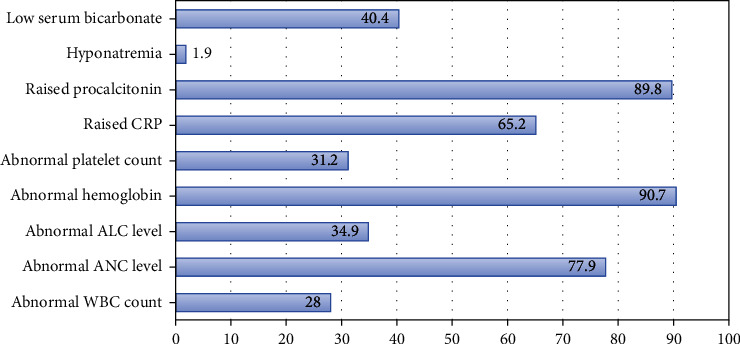
Laboratory investigation results in pediatric patients studied (*n* = 405).

The basic laboratory parameters collected near universally for ill children presenting to the emergency department were analyzed. Certain advanced tests such as procalcitonin, D-dimer, ferritin, and fibrinogen were not included in the current study given the scarce utilization of such higher tier investigations in the primary wave of the pandemic. As Figure 1 demonstrates, the most frequently observed laboratory features in studied population were low hemoglobin (90.7%), raised procalcitonin (89.8%), and abnormal (low or high) absolute neutrophil count (77.9%). Each individual laboratory parameter was subsequently studied in relation to COVID-19 swab outcomes. The patients with negative test demonstrated higher percentage of raised inflammatory markers (c-reactive protein and procalcitonin) compared to those with positive results (67.7% versus 38.1%; 93.3% versus 72.7%). The differences were statistically significant (*p* < 0.05).

### 3.5. Chest X-Ray Features

Radiologic imaging findings were broadly grouped into three categories upon basis of commonality in the studied population: normal, nonspecific infiltrates, and lobar consolidation. It was found that the highest percent of the patients demonstrated nonspecific infiltrates (64.4%). Each individual chest X-ray finding was subsequently studied in relation to COVID-19 swab outcomes; patients with positive COVID-19 test showed a higher percent of nonspecific infiltrates than those of negative test (80% versus 63.3%). No statistically significant difference could be ascertained. Advanced imaging modalities such as computed tomography scans of the lungs were not in practice as per routine in suspected patients, hence not included in the study

### 3.6. Logistic Regression Analysis of Factors Associated with Positive COVID-19 Test

Patients with history of contact with COVID-19 cases are more likely to be COVID-19 positive than those without such history (OR = 12.20, *p* < 0.05). Patients with fever and cough had double the risk of having positive test relative to those with fever or runny nose (*p* < 0.05). Higher nonsignificant risk was observed among those with lymphadenopathy (OR = 4.08) (Table 1). Patients with hyponatremia were more likely to be COVID-19 test positive with almost double the risk relative to those with normal sodium level. Regarding the result of chest X-ray, those with and nonspecific chest X-ray infiltrates and lobar consolidation had higher risk of getting positive relative to those with normal X-ray (OR = 2.93 and 3.87, respectively; *p* > 0.05) (Table 2).  Table 3 presents the stepwise logistic regression for the predictors of positive COVID-19 test. It was found that history of contact and C reactive protein lead to a high significant risk of about 18 times to have positive test relative to those without such features

## 4. Discussion/Conclusion

Published reports from different countries over 2020 have shown that pediatric patients represent a marginal proportion of COVID-19 cases (less than 2% of total reported) [15, 20]. Our findings are like these data, as just a minority (8%) of the study population tested positive over first eight months of the pandemic. Several hypotheses have been proposed to explain milder disease nature in children, such as trained immunity secondary to routine immunization and immaturity of angiotensin-converting enzyme receptor 2 which is necessary for virus binding [16, 21].

Increasing reports seem to suggest that infants under the age of 1 year and those with underlying comorbidities may be at increased risk of severe illness from SARS-CoV-2 infection [15]. Our study included both aforementioned demographics, yet fortunately upon the bases of initial emergency department presentations, there was no convincing evidence of severe COVID-19 disease.

The main reported associated factors for the pediatric COVID-19 infections are close contact with a family member with an infection and/or a history of travel or residence in an endemic area [22]. Our study has mirrored these observations by demonstrating a statistically significant seventeen (17) times higher fold risk of COVID-19-positive test in children who were reportedly exposed to an infected individual.

Among the commonly reported symptoms in children aged ≤9 years are fever (46%) and cough (37%) [23]. Our study demonstrated the commonest presenting complaints included fever (41.7%), vomiting/diarrhea (31.9%), and cough (21.5%). Of these, there is a statistically significant difference between presence of a runny nose, cough, poor feeding, and abdominal pain to positive COVID-19 nasal swab result (*p* < 0.05).

Physical examination findings of congested ear/nose/throat (15.3%), dehydration (13.1%), and tenderness on palpation of the abdomen (11.4%) were demonstrated in our overall study population; however, most COVID-19-positive cases had minimal examination features correlating with a mild upper respiratory tract infection. As in adults, children with severe COVID-19 may develop respiratory failure, myocarditis, shock, acute renal failure, coagulopathy, and multiorgan system failure [24], but fortunately none of our study population COVID-19 positive patients exhibited such severe features.

Investigation-wise, nucleic acid detections using reverse transcriptase real-time polymerase chain reaction qualitative or quantitative are the commonest methods for diagnosis [25], as is used in our hospital facility. Identical to our findings, reports on COVID-19-positive cases suggest white blood cell count normal or reduced, C-reactive protein normal or increased, and procalcitonin normal on most occasions [23, 26, 27]. High tier laboratory investigations such as liver enzymes, muscle enzymes, myoglobin, ferritin, fibrinogen, interleukins, coagulation studies, cardiac enzymes, and D-dimer may be elevated in severe cases; however, testing for them is not part of routine investigations for mildly ill children and hence not included in this study.

Chest X-rays in pediatric COVID-19 infections are often reported as either normal or with nonspecific viral pneumonia-like changes [15]. Our study demonstrated a parallel observation of 80% COVID-19-positive children having nonspecific infiltrates.

The present study bears certain limitations as it is retrospectively conducted; hence, data is heavily reliant upon accuracy of physician assessment, documentation, and quality of obtaining COVID-19 nasal swab.

In conclusion, our study has demonstrated that only a minority of the studied pediatric population (eight percent) has tested positive for COVID-19 infection in the first eight months of the pandemic. This research has shown that the COVID-19-positive pediatric population exhibits parallel findings to international reports regarding: exposure to COVID-19-positive individuals with high significant risk of about eighteen times, frequent presence of fever, and cough, with the associated of almost double the risk of getting positive, reassuring physical examination findings, and predominantly normal reports of basic blood investigations in addition to abnormal findings in chest X-ray images as nonspecific infiltrations and lobar consolidation.

It is recommended to invest in continuation of similar pediatric research studies over the coming year of 2021 in order to understand the clinical evolution of subsequent COVID-19 infection waves and the potential impact of mass vaccination on the picture of COVID-19 infection in children.

## Figures and Tables

**Table 1:** Univariate logistic regression of the associated factors (characteristics, presenting symptoms, and physical examination findings) of positive COVID-19 test.

Independent variables	Categories (total no.)	Positive COVID-19 test	*p*	OR	95% confidence interval	
		*N* = 32 No. (%)			Lower	Upper
Characteristics						
Age (years)	<5 (*n* = 160) 5+ (*n* = 245)	22 (9.0) 10 (6.3)	0.322	1.48	0.68	3.21
Gender	Male (*n* = 220) Female (*n* = 185)	17 (7.7) 15 (8.1)	0.887	1.05	0.51	2.17
Nationality	Local (*n* = 146) Nonlocal (*n* = 259)	10 (6.8) 22 (8.5)	0.556	1.26	0.58	2.75
Comorbidity	Absent (*n* = 220) Present (*n* = 185)	18 (8.2) 14 (7.6)	0.819	0.92	0.44	1.90
History of COVID-19 contact	Absent (*n* = 326) Present (*n* = 79)	10 (3.1) 22 (27.8)	0.001^∗^	12.20	5.49	27.12
Presenting symptoms						
Fever	Present (*n* = 169) Absent (*n* = 236)	19 (11.2) 13 (5.5)	0.039^∗^	2.17	1.04	4.53
Runny nose	Present (*n* = 49) Absent (*n* = 356)	11 (22.4) 21 (5.9)	0.001^∗^	1.30	0.51	3.32
Cough	Present (*n* = 87) Absent (*n* = 318)	12 (13.8) 20 (6.3)	0.025^∗^	2.38	1.12	5.09
Poor feeding	Present (*n* = 64) Absent (*n* = 341)	1 (1.6) 31 (9.1)	0.073	0.16	0.02	1.18
Breathing difficulty/apnea	Present (*n* = 42) Absent (*n* = 363)	4 (9.5) 28 (7.7)	0.681	1.26	0.42	3.78
Vomiting/diarrhea	Present (*n* = 129) Absent (*n* = 276)	7 (5.4) 25 (9.1)	0.207	0.58	0.24	1.37
Abdominal pain	Present (*n* = 64) Absent (*n* = 341)	1 (1.6) 31 (9.1)	0.040^∗^	0.16	0.02	1.18
Head/throat/body pain	Present (*n* = 72) Absent (*n* = 333)	7 (9.7) 25 (7.5)	0.528	1.32	0.55	3.19
Physical examination findings						
Congested ENT	Present (*n* = 62) Absent (*n* = 343)	6 (9.7) 26 (7.6)	0.574	1.30	0.51	3.31
Dehydration	Present (*n* = 53) Absent (*n* = 352)	2 (3.8) 30 (8.5)	0.246	0.42	0.09	1.82
Lymphadenopathy	Present (*n* = 8) Absent (*n* = 397)	2 (25) 30 (7.6)	0.094	4.08	0.79	21.09
Wheezes/crackles	Present (*n* = 35) Absent (*n* = 370)	2 (5.7) 30 (8.1)	0.618	0.69	0.16	3.00
Abdominal tenderness	Present (*n* = 46) Absent (*n* = 359)	1 (2.2) 31 (8.6)	0.159	0.24	0.03	1.77
Skin rash	Present (*n* = 23) Absent (*n* = 382)	1 (4.3) 31 (8.1)	0.523	0.52	0.07	3.95
Joint/tissue swelling	Present (*n* = 14) Absent (*n* = 391)	2 (14.3) 30 (7.7)	0.377	2.01	0.43	9.38

**Table 2:** Univariate logistic regression analysis of the associated factors (laboratory parameters and chest X-ray features) of positive COVID-19 test.

Independent variables	Categories (total no.)	Positive COVID-19 test	*p*	OR	95% confidence interval	
		*N* = 32 No. (%)			Lower	Upper
Laboratory findings						
White cell count	Normal (*n* = 270) Abnormal (*n* = 105)	23 (8.5) 7 (6.7)	0.554	0.77	0.32	1.85
Absolute neutrophil count	Normal (*n* = 81) Abnormal (*n* = 285)	10 (12.3) 20 (7.0)	0.128	0.54	0.24	1.19
Absolute lymphocyte count	Normal (*n* = 239) Abnormal (*n* = 128)	21 (8.8) 9 (7.0)	0.559	0.79	0.35	1.77
Hemoglobin level	Normal (*n* = 35) Abnormal (*n* = 340)	2 (5.7) 28 (8.2)	0.603	1.48	0.34	6.49
Platelet count	Normal (*n* = 258) Abnormal (*n* = 117)	22 (8.5) 8 (6.8)	0.577	0.79	0.34	1.83
C-reactive protein	Normal (*n* = 87) Abnormal (*n* = 163)	13 (14.9) 8 (4.9)	0.009^∗^	0.29	0.12	0.74
Procalcitonin	Normal (*n* = 13) Abnormal (*n* = 114)	6 (46.2) 16 (14.0)	0.007^∗^	0.19	0.06	0.64
Sodium level	Normal (*n* = 313) Low (*n* = 6)	30 (9.6) 1 (16.7)	0.568	1.89	0.21	16.69
Bicarbonate	Normal (*n* = 190) Low (*n* = 129)	17 (8.9) 14 (10.9)	0.573	1.24	0.59	2.61
Chest X-ray features						
Chest X-ray findings	Normal (*n* = 42)	1 (10.0)	Reference			
	Nonspecific	8 (80.0)		2.93	0.17	
	Infiltrates (*n* = 103)		0.458			49.99
	Lobar consolidation (*n* = 15)	1 (10.0)	0.189	3.87	0.51	29.12

^∗^ *P* < 0.05.

**Table 3:** Stepwise multiple logistic regression of factors affecting positive COVID-19 results.

Independent variables	*B*	*p*	Adjusted odds ratio	95% confidence interval	
				Lower	Upper
Positive COVID-19 contact history	2.863	0.000	17.51	4.59	66.79
C-reactive protein	-1.786	0.007	0.17	0.05	0.62
Constant	-1.050	0.001	0.350		

Model chi‐square = 29.13; *p* = 0.000.

## Data Availability

All data generated or analyzed during this case are included in this article. Further enquiries can be directed to the corresponding author.

## Abbreviations

UAE: United Arab Emirates
LWCH: Latifa Women and Children Hospital
ED: Emergency Department
COVID-19 positive: SARS-CoV-2 virus infection
RT-PCR: Reverse transcriptase real-time polymerase chain reaction
CRP: C-reactive protein
FBC: Full blood count
ANC: Absolute neutrophil count
ALC: Absolute lymphocyte count
ICU: Intensive care unit
N: Number
SIQR: Standard interquartile range.
